# The impact of obesity on sepsis mortality: a retrospective review

**DOI:** 10.1186/1471-2334-13-377

**Published:** 2013-08-16

**Authors:** Ethan F Kuperman, John W Showalter, Erik B Lehman, Amy E Leib, Jennifer L Kraschnewski

**Affiliations:** 1 University of Iowa Hospitals and Clinics, Carver College of Medicine, 200 Hawkins Drive, Iowa City, IA 52242, USA; 2 University of Mississippi Medical College, Oxford, MS, USA; 3 Penn State Milton S. Hershey Medical Center, Penn State College of Medicine, 500 University Drive, PO Box 850, Hershey, PA 17033-0850, USA

**Keywords:** Obesity, Sepsis, Mortality, Diabetes mellitus, Inpatients, Body mass index

## Abstract

**Background:**

Recent sepsis guidelines have focused on the early identification and risk stratification of patients on presentation. Obesity is associated with alterations in multiple inflammatory regulators similar to changes seen in sepsis, suggesting a potential interaction between the presence of obesity and the severity of illness in sepsis.

**Methods:**

We performed a retrospective chart review of patients admitted with a primary billing diagnosis of sepsis at a single United States university hospital from 2007 to 2010. Seven hundred and ninety-two charts were identified meeting inclusion criteria. Obesity was defined as a body mass index (BMI) ≥ 30 kg/m^2^. The data recorded included age, race, sex, vital signs, laboratory values, length of stay, comorbidities, weight, height, and survival to discharge. A modified APACHE II score was calculated to estimate disease severity. The primary outcome variable was inpatient mortality.

**Results:**

Survivors had higher average BMI than nonsurvivors (27.6 vs. 26.3 kg/m2, p = 0.03) in unadjusted analysis. Severity of illness and comorbid conditions including cancer were similar across BMI categories. Increased incidence of diabetes mellitus type 2 was associated with increasing BMI (p < 0.01) and was associated with decreased mortality, with an odds ratio of 0.53 compared with nondiabetic patients. After adjusting for age, gender, race, severity of illness, length of stay, and comorbid conditions, the trend of decreased mortality for increased BMI was no longer statistically significant, however diabetes continued to be strongly protective (odds ratio 0.52, p = 0.03).

**Conclusions:**

This retrospective analysis suggests obesity may be protective against mortality in septic inpatients. The protective effect of obesity may be dependent on diabetes, possibly through an unidentified hormonal intermediary. Further prospective studies are necessary to elaborate the specific mechanism of this protective effect.

## Background

Despite recent improvements in identification and treatment, sepsis remains a common, costly, and deadly condition at U.S. hospitals. With an annual case rate of 3.0 per 1000 population, sepsis costs the U.S. healthcare system an estimated $16.7 billion annually [1]. Additionally, the mortality rate from severe sepsis has risen from 66.8 to 132 per 100,000 population per year between 1993 and 2003 [2]. Despite this increase, earlier recognition and more aggressive treatment of severe sepsis within the first hours of hospital arrival have decreased mortality per admission from 45.8 to 37.8% [2]. These findings underscore the need for rapid identification of patients with severe sepsis and poor prognosis in order to initiate aggressive treatment within the first minutes of hospital arrival.

The obese population may exhibit an altered response to the acute inflammatory stressor of sepsis [3]. The pathologic response in sepsis is mediated through inflammatory cytokines [4], and comparable changes are seen in otherwise healthy patients with obesity [5-8]. Murine models of sepsis have demonstrated increased mortality in obese mice [9,10]. Recent investigations into the alterations in response to sepsis in these animal models have focused on hormonal mediators, including leptin and adiponectin [11-13]. Serum levels of these hormones are also altered in obese humans [14,15]. Furthermore, alterations in serum leptin [16] and adiponectin [17] have correlated with human survival in sepsis, although these relationships are complex and the investigations have been small. These findings suggest a potential correlation between sepsis mortality and obesity, and previous investigators have noted obese patients have impaired immune responses to viral and bacterial pathogens [18].

Despite this potential link, relatively few clinical studies have looked at the clinical interaction. A recent study of bacteremic patients by Huttunen et al. [19] found increased mortality in obese patients, but only 10 of 19 deceased patients had recorded body mass index (BMI) data. Wurzinger et al. [20] found a correlation between increasing BMI and decreased mortality for adult patients with septic shock, but morbid obesity was underrepresented in their sample (only 9 of 303 subjects had BMI > 40 kg/m^2^). In contrast, Nasraway et al. [21] found morbid obesity was significantly associated with death only in the subset of prolonged stay patients (≥ 4 days), but the study followed all surgical intensive care unit admissions and did not report specific information about sepsis. Dossett et al. [22] demonstrated increased survival in morbidly obese patients (87%) compared with patients with normal (84%) or underweight (78%) BMIs in two surgical intensive care units. However the trend failed to reach statistical significance. A recent review of the impact of obesity on pulmonary infections found evidence of increased incidence, severity, and death as a result of the H1N1 influenza pandemic of 2009 among obese patients, but found conflicting results for mortality in bacterial pneumonia [23]. Due to the limitations of currently published research there have been several recent calls for further study to clarify the relationship between obesity and mortality in sepsis [23-26]. The objective of this study was to determine the association between BMI and survival in patients admitted with a diagnosis of sepsis. Based on the limited clinical data, we predicted increased survival in obese and morbidly obese patients.

## Methods

The study was conducted at Penn State Milton S. Hershey Medical Center, an integrated health system including a 473-bed academic medical center, an emergency department with greater than 50,000 annual visits, and an outpatient medical group with over 800,000 visits. A retrospective chart review was performed on all adult patients (≥ 18 years old) who presented to the emergency department or were directly admitted to the hospital with a primary billing diagnosis of sepsis (ICD-9 codes 38.0-38.9) between July 1, 2007 and June 30, 2010. Patients without a documented height and weight (to calculate BMI) were excluded from analysis. The patients’ charts were accessed using a report writing tool that allowed automated extraction of study data from the clinical database. Data points that could not be collected via the clinical database were gathered through manual chart review by a collaborator not directly involved in data analysis. The data recorded included date and time of patient arrival, age, race, sex, vital signs, laboratory values, length of stay, comorbidities, weight, height, and survival to discharge. The primary outcome variable was inpatient mortality. This study was approved by the Penn State College of Medicine Institutional Review Board.

The covariates in this study included BMI, age, race (categorized as white, black, or other), gender, length of stay, comorbid conditions (diabetes mellitus, neutropenia, cancer, liver disease, cardiovascular disease, chronic obstructive pulmonary disease, renal disease, and immunosuppression),and a modified APACHE II score. Modified APACHE II score was used as a measure of disease severity and was based on patient vital signs, mean arterial pressure, comprehensive metabolic panel, oxygenation (based on oxygen saturation and oxygen therapy), arterial pH, mental status change on admission, age, and presence of chronic diseases [27]. The first recorded value within twenty-four hours was used for each category of the APACHE II. The Glasgow Coma Score used in the APACHE II scoring system was approximated using mental status on admission. No mental status change received a score of 15/15, a mild change received a score of 13, and severe change received a score of 10. Similar electronic approaches have been shown to correlate strongly with APACHE II scores [27]. Diagnoses of diabetes and neutropenia were determined based upon ICD-9 codes. All other comorbid conditions were determined from admission documentation.

Weight category was determined by calculating body mass index (BMI) Body Mass Index was divided into five categories (Underweight: < 18.5, Normal: 18.5-24.9, Overweight: 25.0-29.9, Obese: 30.0-39.9, Morbidly obese: 40.0-49.9 kg/m2). Patients with BMI greater than 50 or missing BMI were excluded as outliers (n = 15, range 50.4 to 93.9 kg/m2). Categorical variables were summarized with frequencies and percentages while continuous variables were summarized with means, standard deviations, medians, and quartiles. The distribution of continuous variables was checked using box plots, histograms, and normal probability plots. It was found that length of stay had a skewed distribution, with a minority of patients having a very long length of stay. Therefore, a log transformation was applied to this variable to allow for a comparison by BMI categories with parametric methods. Comparisons between BMI categories were made for continuous variables such as age, length of stay, and APACHE II score using an Analysis of Variance (ANOVA) with the differences quantified by means (Table 1). Comparisons between BMI categories were made for categorical variables using two approaches, a Chi-square test for variables like gender and race and a Cochran-Armitage Trend test for variables like mortality, diabetes, and other comorbidities with differences quantified by percentages (Table 1). Comparisons by mortality of this same set of variables including BMI, as a continuous variable, were made using logistic regression and were quantified with odds ratios (Table 2). All variables were then included as covariates with BMI in a multivariable logistic regression model which adjusts each variable and its accompanying odds ratios for all of the other variables in the model (Table 2). Covariates were analyzed for multicollinearity using the variance inflation factor, and the model fit was checked with the Hosmer and Lemeshow Goodness-of-Fit test. All analyses were conducted using SAS Software version 9.3 (SAS Institute, Cary, NC).

**Table 1:** Variables by body mass index categories

**Variable**	**All**	**Body mass index**					**P-value**
		**Underweight**	**Normal weight**	**Overweight**	**Obese**	**Morbidly obese**	
Subjects, N (%)	792 (100)	49 (6)	261 (33)	249 (31)	187 (24)	46 (6)	
Age +							
Years	61.9 ± 17.2	60.3 ± 22.9	61.2 ± 19.2	64.2 ± 16.0	60.8 ± 14.2	60.2 ± 13.7	0.17
Gender *							
Female	362 (46)	32	106	105	85	34	< 0.01
Male	430 (54)	17	155	144	102	12	
Race *							
White	726 (92)	43	247	221	174	41	0.03
Black	41 (5)	2	11	14	9	5	
Other	24 (3)	4	3	13	4	0	
Length of stay †							
Days	9.1 ± 8.2	8.1 ± 7.2	9.3 ± 8.5	8.8 ± 7.8	9.0 ± 8.1	10.2 ± 9.6	0.64
APACHE II Score +							
Points	15.5 ± 5.9	16.2 ± 6.6	15.4 ± 5.8	15.6 ± 5.8	15.9 ± 6.1	14.2 ± 5.6	0.42
Mortality ‡							
Yes	129 (16)	12	46	40	25	6	0.06
No	663 (84)	37	215	209	162	40	
Diabetes ‡							
Yes	190 (24)	10	17	21	35	48	< 0.01
No	602 (76)	90	83	79	75	52	
Cancer ‡							
Yes	356 (45)	33	46	50	44	24	0.40
No	436 (55)	67	54	50	56	76	
Neutropenia ‡							
Yes	44 (6)	2	5	8	4	9	0.50
No	748 (94)	98	95	92	96	91	
Liver ‡							
Yes	43 (5)	4	5	6	4	9	0.61
No	749 (95)	96	95	94	96	91	
Cardiovascular Disease ‡							
Yes	188 (24)	24	21	24	25	28	0.33
No	604 (76)	76	79	76	75	72	
COPD ‡							
Yes	80 (10)	8	7	10	16	9	0.04
No	712 (90)	92	93	90	84	91	
Renal ‡							
Yes	30 (4)	4	6	3	2	4	0.14
No	762 (96)	96	94	97	98	96	
Immunosuppression ‡							
Yes	78 (10)	16	10	8	11	7	0.29
No	714 (90)	84	90	92	89	93	

*Categorical data presented as N (%) or % and analyzed with Pearson Chi-square test (Exact test if necessary).

+Numeric data presented as Mean ± SD and analyzed with Analysis of Variance.

†Numeric data presented as Mean ± SD, log-transformed for analysis by Analysis of Variance.

‡Categorical data presented as N (%) or % and analyzed with Cochran-Armitage Trend test.

**Table 2:** Variables by mortality**

**Variable (N = 792)**	**Mortality**		**Unadjusted odds ratio (95% CI)**	**Adjusted odds ratio (95% CI)**	**Adjusted P-value**
	**Yes (%)**	**No (%)**			
Age* (OR per 5 years)					
Years	69.8 ± 14.3	60.4 ± 17.3	1.04 (1.02, 1.05)	1.12 (1.04, 1.21)	< 0.01
Gender					
Female	17	83	1.04 (0.71, 1.52)	0.96 (0.63, 1.46)	0.85
Male	16	84	1.0 (reference)	1.0 (reference)	
Race					
White	17	83	1.0 (reference)	1.0 (reference)	
Black	12	88	0.69 (0.21, 1.83)	1.09 (0.39, 3.08)	0.87
Other	12	88	0.71 (0.13, 2.45)	0.92 (0.24, 3.56)	0.90
Length of stay* (OR per 1 day)					
Days	8.6 ± 9.5	9.1 ± 7.9	0.99 (0.97, 1.02	0.99 (0.96, 1.02)	0.65
APACHE II score* (OR per 1 point)					
Points	19.9 ± 6.7	14.7 ± 5.4	1.16 (1.12, 1.20)	1.14 (1.10, 1.19)	< 0.01
Body Mass Index*, kg/m^2^					
kg/m^2^	26.3 ± 6.8	27.6 ± 6.7	0.97 (0.94, 1.0)	0.90 (0.76, 1.06)	0.19
Diabetes					
Yes	11	89	0.53 (0.32, 0.88)	0.52 (0.29, 0.93)	0.03
No	18	82	1.0 (reference)	1.0 (reference)	
Cancer					
Yes	16	84	0.96 (0.66, 1.41)	1.03 (0.64, 1.65)	0.90
No	17	83	1.0 (reference)	1.0 (reference)	
Neutropenia					
Yes	9	91	0.50 (0.18, 1.42)	0.40 (0.13, 1.24)	0.11
No	17	83	1.0 (reference)	1.0 (reference)	
Liver					
Yes	28	72	2.09 (1.04, 4.19)	1.68 (0.75, 3.78)	0.21
No	16	84	1.0 (reference)	1.0 (reference)	
Cardiovascular Disease					
Yes	22	78	1.71 (1.13, 2.58)	0.91 (0.55, 1.52)	0.73
No	14	86	1.0 (reference)	1.0 (reference)	
COPD					
Yes	18	82	1.10 (0.60, 2.03)	0.79 (0.40, 1.54)	0.48
No	16	84	1.0 (reference)	1.0 (reference)	
Renal					
Yes	17	83	1.03 (0.30, 2.81)	0.96 (0.32, 2.87)	0.94
No	16	84	1.0 (reference)	1.0 (reference)	
Immunosuppression					
Yes	15	85	0.93 (0.49, 1.77)	0.79 (0.39, 1.63)	0.53
No	16	84	1.0 (reference)	1.0 (reference)	

*Categorical data presented as Percent, Numeric data presented as Mean ± SD.

**Logistic regression used for all analyses, Adjusted odds ratios are adjusted for all variables in the table.

## Results

Chart review identified 845 patients with a presenting diagnosis of sepsis over the specified time period. Of these patients, 792 met inclusion and exclusion criteria. There were 129 inpatient deaths in the study population. Demographics of the cohort by BMI category are summarized in Table 1. Six percent of study participants were morbidly obese, 24% were obese and 31% were overweight, comparable to recent national statistics [28]. Septic patients who were underweight or morbidly obese were significantly more likely to be female. As expected, the prevalence of diabetes mellitus increased significantly with increasing BMI. There were also statistically significant differences in race across BMI categories. Length of stay (average 6.5 days) and disease severity, measured by modified APACHE II score, were not significantly associated with alterations in BMI.

Using a Cochran-Armitage test, increased mortality trended towards a lower BMI category, although the trend failed to reach statistical significance (p = 0.06). Using normal weight (BMI 18.5-24.9 kg/m^2^) as a reference group in logistic regression, the odds ratio for mortality was 1.5 (95% CI 0.67-6.3) for underweight patients and 0.7 (95% CI 0.12 – 4.2) for those with morbid obesity (p = 0.19). This clinically significant variation in mortality with respect to BMI reached statistical significance in the analysis using BMI as a continuous variable. Survivors had a BMI of 27.6 kg/m^2^, compared with 26.3 kg/m^2^ among non-survivors (p = 0.03).

This difference in mortality was not explained by differences in comorbid conditions. Comorbidities across BMI categories are summarized in Table 1. There were similar prevalences of cancer and neutropenia between BMI categories. Of the comorbid conditions examined for this study, only chronic obstructive pulmonary disease (COPD, p = 0.04) and diabetes mellitus (p < 0.01) were found to be associated with BMI category. Table 2 summarizes the relationships between comorbid conditions and inpatient mortality for this cohort. The odds ratio for mortality comparing patients with and without COPD was 1.10 (95% CI 0.6 to 2.03, p = 0.76), failing to reach statistical significance. Pre-existing liver and cardiovascular diseases were found to significantly predispose to inpatient mortality but did not correlate with BMI. Diabetes mellitus was found to be significantly protective against death, with an odds ratio for mortality of 0.53 (95% CI 0.32-0.88, p = 0.01). Due to the high mortality and low incidence of diabetes mellitus in patients with low BMI, the analysis was repeated excluding underweight (BMI < 18.5 kg/m^2^). The results were unchanged, with an OR for mortality of 0.54 (95% CI 0.32-0.91, p = 0.02).

To better characterize the correlation between obesity and survival, multivariate analysis was performed using the tabulated demographic variables and comorbid conditions (Table 2). Race and COPD, which had been found to correlate with BMI, were not associated with significant changes in mortality. Increasing age (per 5 year increments) and increasing APACHE II score (per 1 point) were both found to be independent risk factors for increased mortality (adjusted OR 1.12 and 1.14 respectively, p < 0.01). When adjusted for the presence of diabetes, BMI trended towards protection from inpatient mortality (OR 0.90 for 5 kg/m^2^ increase, p = 0.19) but failed to reach statistical significance. Diabetes mellitus remained strongly protective with OR 0.52 (95% CI 0.29-0.93, p = 0.03) despite adjustment for other comorbid conditions. No other comorbidity was found to have a statistically significant relationship with inpatient survival.

## Discussion

Obesity, including morbid obesity, was protective for inpatient sepsis mortality within this cohort, but the protection appears to have been due to the presence of comorbid insulin resistance and diabetes. This finding, although similar to the results of previous clinical trials [20-22] is not easily explained through our current understanding of the physiology of obesity, diabetes, and sepsis. One possible mediator of this protection is leptin, a hormone highly elevated in human obesity [15]. Leptin-knockout mice have excess weight gain and have been used as an *in vivo* model of human obesity. When exposed to a septic challenge, mice incapable of secreting leptin have increased serum levels of TNF- α and IL-6 as well as increased mortality [9,10,29]. This increase in mortality is partially reversed by treatment with exogenous leptin [9,10]. Leptin has also been shown to be independently elevated by critical illness in non-obese humans [16] and to reduce endotoxin-induced TNF-α production [29].

Diabetes mellitus has been associated with increased incidence of infectious disease and increased infectious disease mortality [30-32], however in this cohort the presence of diabetes was associated with markedly reduced mortality. A previous study by Whitcomb et al. [33] found a similar trend towards decreased mortality for septic patients with a previous diagnosis of diabetes despite increased mortality for patients with newly-diagnosed hyperglycemia. Other previous attempts to prove an association between diabetes mellitus and sepsis mortality have had equivocal results. Two studies observed no difference in mortality between diabetic and nondiabetic patients with sepsis despite diabetic patients having increased comorbidities at admission [34,35]. Diabetes mellitus has also been associated with decreased acute lung injury in severe sepsis [36]. This suggests diabetic patients may have increased incidence of sepsis or present with more severe illness, but are less likely to die from an individual episode of sepsis when controlling for disease severity. The mechanism for this protection is unclear, and may be due either to the pathology and hormonal changes of diabetes or the medical therapy used to treat it.

Animal models attempting to separate the effects of diabetes and obesity in sepsis response have been limited. The Akita mouse model develops severe diabetes mellitus through a mutation in the Ins2 gene leading to accumulation of proinsulin and β-cell dysfunction [37]. In contrast to the results of this study, Akita mice had increased mortality and decreased pro-inflammatory cytokines compared with wild type controls when under septic challenge [38]. However, the diabetes in these mice was untreated and it is unclear if the results can be compared with this human population where both diabetes and sepsis were treated aggressively. Diabetes mellitus can also be induced in mice by exposure to streptozotocin, an antibiotic with β-cell toxicity [39]. Mice with streptozotocin-induced diabetes exhibit increased mortality and decreased bacterial clearance compared with controls after receiving a septic challenge, which partially corrected after being treated with insulin [40]. However, both models would exhibit insulin deficiency, and would have significantly different adipokine levels in comparison with the diabetic patients in this study population.

Previous efforts to link tight glycemic control with sepsis survival in humans have been complicated by high rates of hypoglycemia leading to early termination [41]. If endocrine mediators are responsible, pharmacologic modification of serum hormone levels may alter sepsis mortality. For example, increased serum levels of adiponectin are associated with sepsis survival, and in animal models these levels are increased by exposure to rosiglitazone (an oral medication used to treat diabetes) resulting in improved survival with a septic challenge [42]. Unfortunately an accurate record of the patients’ home medications was not contained within the electronic medical record for this cohort. Further elucidation of the mechanism of protection may lead to improved outcomes not only in the diabetic patients but also in the nondiabetic, nonobese population through pharmacologic manipulation of inflammatory regulation.

The underweight (BMI < 18.5 kg/m^2^) population had increased inpatient mortality compared with the overall cohort (24% vs. 16%). Previous investigators have found increased incidence of sepsis and severe sepsis in the underweight population [43] as well as higher disease severity at admission [44]. The trend towards worsened survival in this cohort does not appear to be due to increased incidence of comorbid conditions including cancer, renal disease, or liver disease as these conditions were not present at higher rates in patients who were underweight. Whether the observed statistical trend was due to loss of physiologic reserve, the presence of other comorbidities not captured on our retrospective review, or the continuation of physiologic processes protective in obese patients cannot be ascertained without further prospective investigation.

## Conclusions

This study is one of the largest examining the relationship between BMI, diabetes, sepsis and mortality. Strengths of this study include the large cohort size and the use of an electronic medical record to capture a nearly complete database of the patients selected for study inclusion. The multivariate analysis including common comorbid conditions, and the strength of the relationship between diabetes and decreased mortality was robust even after adjusting for numerous covariates.

There were several limitations to this study. The results of this single-center study will need to be documented at other institutions to ensure generalizability. Due to the limitations of the electronic medical record, BMI was used to define obesity. Additionally, we limited our analysis to patients with a BMI < 50 kg/m^2^, as the small number of patients (n = 15) above this BMI there was insufficient statistical power to draw meaningful conclusions. Therefore, these results cannot be extrapolated to individuals above this BMI range. As a retrospective chart review, we were limited to information documented in the electronic medical record. This impeded analysis of glycemic control and follow-up of patients beyond hospital discharge. Similarly, the etiology of sepsis (pneumonia, bloodstream infection, urinary infection) could not be determined from this data set. It also prevented collection of additional data, such as serum concentrations of leptin or adiponectin, which may help clarify the underlying mechanism of the protection seen in diabetic patients. Further, there was no standardization of treatment for diabetic or hyperglycemic patients in this cohort, and information was unavailable about outpatient pharmacotherapies, including oral hypoglycemic agents taken by patients prior to hospital arrival. Although this study analyzed diabetes mellitus as a categorical variable, in reality diabetes represents a spectrum of disease with variable insulin resistance and blood glucose levels. Further investigations will be required to fully understand this trend.

This study adds to the growing body of evidence that obesity is not universally detrimental to survival, and may be a clinically useful model for underlying protective processes. Although the mechanism of this protection is not understood, it appears to be related to insulin resistance, possibly through endocrine mediators. Given the protective effect against mortality identified in patients with diabetes mellitus, insulin and glucose levels may also strongly affect the immune response. Future prospective studies will be needed to better define this relationship.

## Competing interests

The authors declare they have no competing interests.

## Authors’ contributions

EK assisted with study design, performed literature review, interpreted the results, and drafted the manuscript. JS organized automated collection of chart data, assisted with study design, assisted with statistical analysis and interpretation, and helped draft the manuscript. EL performed statistical analysis and created tables. AL performed manual chart review and helped review the manuscript. JK conceived of the study, participated in its design, and helped draft the manuscript. All authors reviewed the manuscript prior to submission. All authors read and approved the final manuscript.

## Pre-publication history

The pre-publication history for this paper can be accessed here:

http://www.biomedcentral.com/1471-2334/13/377/prepub
